# Effect of Moxibustion on HIF-1*α* and VEGF Levels in Patients with Rheumatoid Arthritis

**DOI:** 10.1155/2019/4705247

**Published:** 2019-11-27

**Authors:** Yuanyuan Gong, Zeyun Yu, Yingni Wang, Yan Xiong, Yumei Zhou, Chen-xi Liao, Yuan Li, Yun Luo, Yu Bai, Bailu Chen, Yuzhi Tang, Ping Wu

**Affiliations:** ^1^Chengdu University of Traditional Chinese Medicine, Sichuan, Chengdu 610075, China; ^2^Dazhou Central Hospital, Sichuan, Dazhou 635000, China; ^3^The Fourth Clinical Medical College of Guangzhou University of Chinese Medicine, No.1 Fuhua Road, Futian District, Shenzhen, GuangDong 518033, China; ^4^The First Teaching Hospital, Chengdu University of Traditional Chinese Medicine, Sichuan, Chengdu 610075, China; ^5^Jiangyou Second People's Hospital, Sichuan, Jiangyou 621702, China; ^6^Department of Acupuncture and Rehabilitation, Hospital (T.C.M) Affiliated to Southwest Medical University, Sichuan, Luzhou 646000, China

## Abstract

**Background:**

Moxibustion has a therapeutic effect of reducing swelling and relieving pain in patients with rheumatoid arthritis (RA) but its mechanism is uncertain.

**Objective:**

To evaluate the effect of moxibustion on serum levels of hypoxia-inducible factor-1*α* (HIF-1*α*) and vascular endothelial growth factor (VEGF) in patients with RA and to explore the possible mechanism of moxibustion.

**Methods:**

This study involved 46 RA patients who had fulfilled the inclusion criteria and were randomly assigned to a treatment group and a control group in an equal ratio. The control group was treated with methotrexate or leflunomide, while the treatment group received methotrexate or leflunomide and moxibustion at ST 36 (*Zusanli*), BL 23 (*Shenshu*), and *Ashi* points. Patients' clinical symptoms, RA-associated serum markers, and serum levels of TNF-*α*, IL-1*β*, HIF-1*α*, and VEGF were compared in the two groups before and after intervention. Statistical analysis was performed using SPSS 21.0 statistical software.

**Results:**

37 of 46 RA patients eventually completed the whole treatment course. Compared with the control group, the treatment group significantly improved the clinical symptoms (*P* < 0.05) but with no significant differences in RA-associated serum markers (*P* > 0.05). There were significant differences in TNF-*α* and IL-1*β* among the groups after 8 weeks of treatment (*P* < 0.05). HIF-1*α* and VEGF were decreased in the treatment group after therapy (*P* < 0.05). VEGF was reduced in the control group (*P* < 0.05), while HIF-1*α* was not significantly improved (*P* > 0.05). The reductions of HIF-1*α* and VEGF in the treatment group were superior to the control group (*P* < 0.05).

**Conclusions:**

Moxibustion enhanced the anti-inflammatory and analgesic effects of conventional medicine and can enhance the effect of conventional medicine, downregulating HIF-1*α*/VEGF contents to inhibit angiogenesis.

## 1. Introduction

Rheumatoid arthritis (RA) is a chronic inflammatory joint disease, which can cause cartilage and bone damage as well as disability [1]. The morbidity of RA is as high as 0.18–1.07% all over the world, which shows an increasing trend year by year [2].

The basic pathogenesis of RA is generally centered around the underlying inflammatory process that affects the synovium of the joint which is commonly associated with angiogenesis, the formation of new blood vessels from the pre-existing vascular network [3, 4]. Angiogenesis can foster the infiltration of inflammatory cells into the joints, is an early occurrence of inflamed joint tissue, and plays a major role in the development of arthritis [5, 6]. The process of synovial cell hyperplasia occurs causing the synovial lining to become thickened, resulting in the joint cavity hypoxia that promotes angiogenesis, forming a pannus, destructing the cartilages and bones, and resulting in pain and dysfunction [7–9]. Conversely, inhibition of joint neovascularization can alleviate synovitis and pannus formation [7]. Studies on related cytokines that affect angiogenesis may be the target of RA treatment.

The vascular endothelial growth factor (VEGF) participates in the regulation and production of new blood vessels by the specific action on vascular endothelial cells, causing a series of signal transduction through binding with its receptor, releasing a variety of growth factors and inflammatory factors, causing endothelial cells to proliferate and migrate, and eventually producing a large number of new blood vessels [10]. RA joint hypoxic environment has a close relationship with excessive formation of the synovial vessels. Hypoxia-inducible factor (HIF) -1*α*, which abundantly expressed under micro-oxygen conditions, contributes to hypoxia-augmented inflammatory cytokine production, promotes angiogenesis, invasion, and metastasis, and with the addition of pro-inflammatory cytokines TNF-*α* and IL-1*β* up-regulates the expression of VEGF under hypoxia to promote angiogenesis [11, 12]. Therefore, HIF-1*α* and VEGF levels in RA patients can reflect the progression of angiogenesis from one aspect.

Rheumatoid arthritis (RA) is considered a chronic disease that cannot be cured [13]. Conventional medicine can only temporarily control the disease and long-term use might have a side effect on the liver and kidney function. Disease-modifying antirheumatic drugs (DMARDs), the key therapeutic agents, can reduce synovitis and systemic inflammation and provide pain relief. In addition, surgery has a certain effect on the functional recovery of some large joints of RA patients, which can suspend the condition of the disease. But the high cost is not suitable for most patients, and surgery cannot prevent disease progression as it does not address the underlying disease pathogenesis.

Moxibustion is a kind of traditional Chinese therapy, which has the advantage of good clinical efficacy and no toxic effects. A study has shown that moxibustion can regulate inflammatory cytokines and VEGF in RA [14], which can also significantly inhibit the proliferation and secretion of RA synovial cells, thus effectively improving the synovial inflammation hyperemia, edema, and joint cavity effusion and having good anti-inflammatory, analgesic, and immunomodulatory effects [15]. In our previous study, we found the contents of HIF-1*α* and VEGF can be regulated by moxibustion ST 36 and BL 23 to inhibit the formation of blood vessels in RA rabbit synovial tissue. Therefore, the aim of this study was to discuss the effect of moxibustion on the levels of angiogenesis-related factors HIF-1*α* and VEGF in RA patients, which can provide a more solid basis for moxibustion in RA treatment.

## 2. Materials and Methods

**Figure 1:**
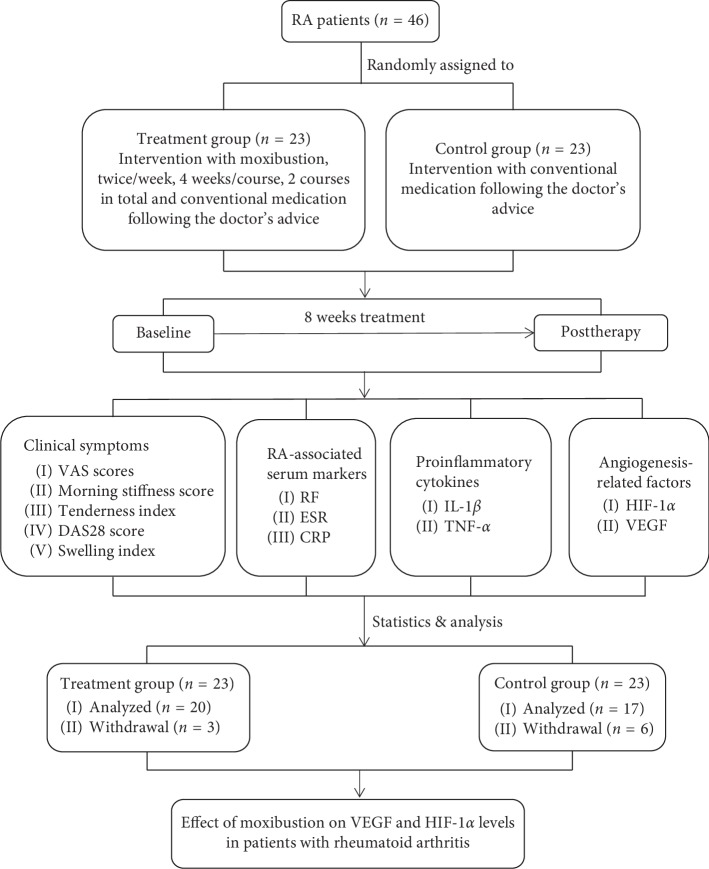
Technology roadmap.

This study was conducted at a Chengdu University of Traditional Chinese Medicine-affiliated hospital from Mar. 2017 to Nov. 2017 (Figure 1). The research ethics approval was obtained prior to the study, and all the participants signed an informed consent.

### 2.1. Participants

All participants were recruited in strict accordance with the following criteria, then randomly assigned to either the treatment or control group in equal ratio. The control group was treated by using methotrexate or leflunomide and the treatment group received moxibustion besides conventional medicine.

#### 2.1.1. Inclusion Criteria

The participants should meet all the following conditions:

1. Diagnosed with RA (according to the ACR/EULAR 2010 criteria [16, 17])
2. Aged between 18 and 65 years, DAS28 > 3.2
3. With clear consciousness and able to cooperate with this study
4. Receive no other antirheumatic drugs within 24 weeks [18]
5. Sign an informed consent for the clinical study

#### 2.1.2. Exclusion Criteria

Patients with any one of the following conditions were excluded from this research:

1. Unconscious and unable to complete the study
2. Advanced patients with severe deformity of joints and the function is in stage IV
3. With other autoimmune diseases, such as systemic lupus erythematosus and Sjogren's syndrome, and mixed connective tissue disease
4. With some severe diseases in various systems or malignant tumor
5. Women in pregnancy or nursing
6. Diagnosed with a psychiatric disorder
7. Allergic to a variety of drugs
8. Be afraid of the moxibustion treatment

#### 2.1.3. Sample Size

Based on similar mechanism studies that have been already performed in domestic and foreign settings, RA human trial requires at least 16–19 participants in each group [18, 19]. Considering a 20% withdrawal rate, the sample size was estimated at more than 20 participants in each group.

#### 2.1.4. Randomization and Blinding

Patients who met the inclusion criteria were recruited. Eligible participants were randomly assigned to either the treatment group or control group in a 1 : 1 ratio via a table of random numbers, which was generated by using SPSS 21.0 statistical software. Opaque envelopes were used to seal the randomization information. The randomization was overseen by an independent researcher.

This study compared the effect of moxibustion therapy with conventional medicine. As it is easy to know whether moxibustion treatment was performed, it is unable to blind the patients and clinical practitioners. However, data collectors and statisticians were blinded in order to eliminate potential bias.

### 2.2. Interventions

**Figure 2:**
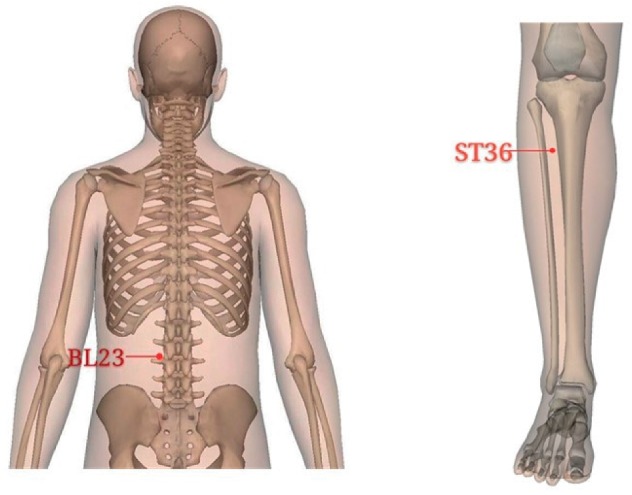
Acupoints. BL23 (Shenshu) is located adjacent to the spinous process of the second lumbar vertebra, ST36 (Zusanli) is on the anterior lateral side of the shank, and “Ashi” points are located at where swelling and paining occur.

All patients received methotrexate (2.5 mg/pill) or leflunomide (10 mg/pill) following the doctor's advice and long-term oral therapy. In addition, patients in the treatment group received moxibustion at bilateral acupoints of BL 23 (*Shenshu*), ST 36 (*Zusanli*), and *Ashi* points, which were located based on the National Standard of the People's Republic of China (GB/T12346-2006) *Acupoint Name and Positioning* (Figure 2). Moxibustion operation was performed 2 times per week, 4 weeks for a course of treatment, with a total of 2 courses. Moxibustion was performed by licensed-TCM doctors with over 3-year experiences of clinical practice.

**Figure 3:**
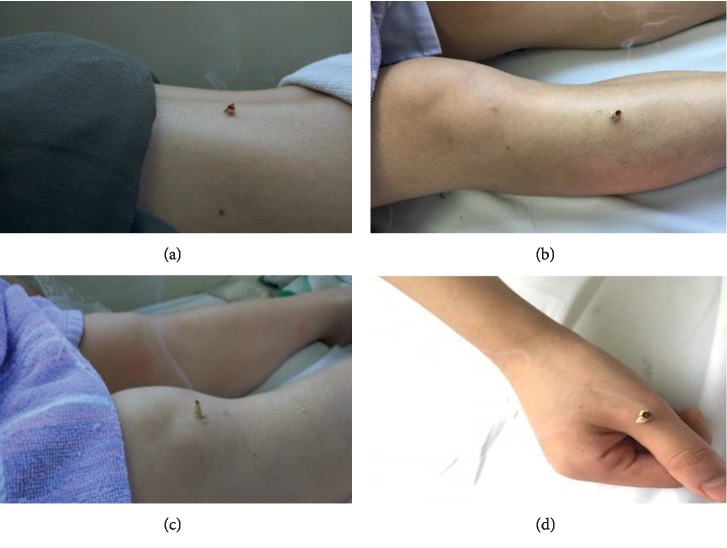
Diagram of moxibustion. (a) Participant was treated at acupoint BL23 (Shenshu). (b) Participant was treated at acupoint ST36 (Zusanli). (c) and (d) Participants were treated at “Ashi” points.

Practitioners labeled the acupoints with a marker and disposed them with Vaseline. The moxa cone was handmade using mugwort floss and put on acupoints and then ignited the top of the moxa cone (Figure 3). The cone was lifted up quickly and put on another one if the patient felt burning pain during the treatment. Five to seven consecutive moxa cones were burned at each point until the local skin of the acupoint blush but no blister was seen.

### 2.3. Outcome Measures

The signs and symptoms utilizing ACR20 and changes of the VAS and the DAS28 score at the baseline and after 8 weeks of treatment were evaluated. The changes in ESR, SCRP, and RF were compared as well. Contents of serum TNF-*α*, IL-1*β*, HIF-1*α* and VEGF in the two groups were compared before and after the treatment. The safety of moxibustion was assessed by the occurrence of adverse events, such as burning and allergy.

### 2.4. Specimen Collection

We collected 3–5 ml of elbow vein blood before and after the patient received treatment and preserved blood serum at a low temperature of−80 degrees. And then, all the serums were sent to the Chengdu LiLai biomedical Experiment Center for the tests of human TNF-*α*, IL-1*β*, HIF-1*α*, and VEGF, which were measured by enzyme-linked immunosorbent assay (ELISA) according to the kit manufacturer's instructions.

### 2.5. Statistical Analysis

Data were analyzed using SPSS 21.0 software (SPSS, Inc., Chicago, Illinois, USA) and expressed as mean and standard deviation. The contents of statistical analysis of both group patients were as follows: (1) general information: name, gender, age, height, and weight; (2) content of HIF-1*α* and VEGF; (3) clinical symptom indexes: VAS score, morning stiffness score, tenderness index, swelling index, and DAS28 score; and (4) RA-associated serum markers: ESR, SCRP, and RF content. Count data used *X*^*2*^ test and normal distribution of the measurement data used a *t-*test, and data in each group were analyzed by using paired-samples *t-*test while the analysis was performed between the two groups using independent samples *t*-test. Non-normal distribution of measurement data used nonparametric test, while the analysis including the two groups used Wilcoxon signed-rank sum test, and the analysis between the two groups used the Mann–Whitney *U* test. A probable value of *P* < 0.05 was considered to be statistically significant.

## 3. Results

### 3.1. Baseline Characteristics

46 patients were included in this study after screening and 23 in each group were randomly assigned to the two groups. In the treatment group, two patients withdrew due to busy time schedule (*n* = 2) and one due to losing interest (*n* = 1); in the control group, four withdrew due to losing interest (*n* = 4) and two due to losing contact (*n* = 2). A total of 37 participants (20 in the treatment group and 17 in the control group) were included in the statistical analysis.

Table 1 shows there were no significant differences in baseline characteristics between the two groups (*P* > 0.05).

### 3.2. The Clinical Symptoms and RA-Associated Serum Markers

Significant differences were noted in VAS scores, morning stiffness score, tenderness index, DAS28 score (*P* < 0.01, *P* < 0.05), and swelling index (*P* < 0.01, *P* < 0.01) between baseline and posttherapy in the two groups, indicating that symptoms were improved after treatment. After 8 weeks, clinical symptoms of the treatment group were significantly improved compared with the control group (*P* < 0.01) (Table 2).

There were significant differences in ESR and CRP contents at baseline and after treatment of both groups (treatment group, *P* < 0.01; control group, *P* < 0.05). The result of RF content revealed that there was a significant difference in the treatment group between baseline and posttherapy (*P* < 0.01). Meanwhile, there was no significant difference in the control group (*P* > 0.05). However, intergroup comparisons did not show significant differences in ESR, CRP, and RF contents after treatment (*P* > 0.05) (Table 2). It indicated that there was no significant difference in the effect of moxibustion and conventional medicine on RA-associated serum markers.

### 3.3. The Contents of TNF-*α* and IL-1*β*

In the treatment group, there were significant decreases in contents of TNF-*α* and IL-1*β* between baseline and after therapy (*P* < 0.01, *P* < 0.01). In the control group, both TNF-*α* and IL-1*β* content showed no significant differences (*P* > 0.05, *P* > 0.05). There were significant differences in TNF-*α* and IL-1*β* among the groups after 8 weeks of treatment (*P* < 0.05) (Table 3).

### 3.4. The Contents of HIF-1*α* and VEGF

**Figure 4:**
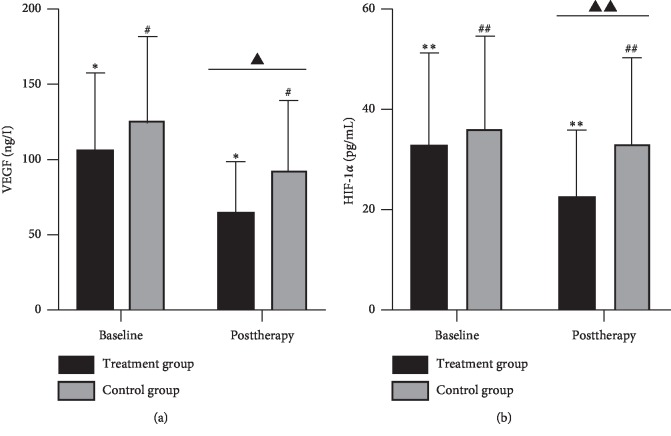
HIF-1*α* and VEGF contents in the serum of two groups. The content of HIF-1*α*, ^*∗∗*^*P* < 0.001, ^##^*P* > 0.05, ^▲▲^*P* < 0.05. The content of VEGF, ^*∗*^*P* < 0.001, ^#^*P* < 0.05, ^▲^*P* < 0.05. Data were expressed as mean (SD).

Both HIF-1*α* and VEGF were decreased in the two groups after treatment. Compared with the control group, the treatment group could significantly reduce the serum levels of HIF-1*α* and VEGF (Table 3, Figure 4).

## 4. Discussion

This study was focused on changes of HIF-1*α* and VEGF contents to evaluate the effect of moxibustion on serum levels of HIF-1*α* and VEGF in patients with RA by comparing with conventional medicine strategy to explore the possible mechanism of moxibustion.

### 4.1. The Changes in Clinical Symptoms and RA-Associated Serum Markers before and after the Treatment

Our results showed that pain and other related symptoms in RA patients can be significantly improved after an 8-week treatment by moxibustion combined with conventional medicine, indicating the effectiveness of moxibustion for treating RA.

However, the RA-associated serum markers did not show significant differences between the two groups. We speculate that it might be because RA is a chronic autoimmune disease and CRP and ESR are the main indicators for the acute reaction of RA inflammation, short-term treatment may not show obvious differences in these indicators. From the clinical symptoms of RA patients, aspects of improvement were obvious but changes in RA-associated serum markers may take longer time to be observed.

### 4.2. The Effect of Moxibustion on TNF-*α*, IL-1*β*, HIF-1*α*, and VEGF in Serum of Patients with RA

The main pathological feature of RA is the inflammation of the synovium. Angiogenesis is the core premise of synovitis continuous reaction and mainly results in the hypoxia state of tissues [20]. Hypoxia leads to the accumulation of HIF-1*α* and finally, induces the expression and secretion of VEGF by macrophages and RA synovial tissue fibroblasts. Moreover, synovial inflammation promotes the production of pro-inflammatory cytokines such as TNF-*α* and IL-1*β*. The pro-inflammatory cytokines together with the increased HIF-1*α* can then activate the production of VEGF [7]. The reduction of TNF-*α* and IL-1*β* indicated moxibustion enhanced the anti-inflammatory effect of conventional medicine.

**Figure 5:**
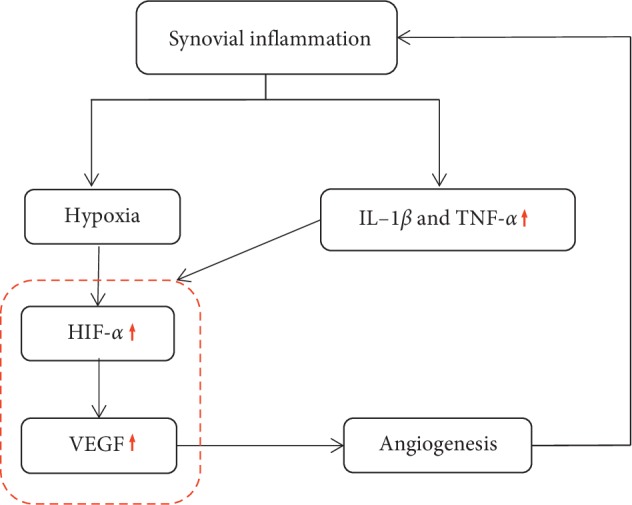
HIF-1*α* and VEGF foster angiogenesis.

VEGF is a key regulator in the formation and maintenance of pannus [21, 22] and abundantly expressed in synovial fluid and serum of RA patients [21, 23, 24]. In general, HIF-1*α* can up-regulate the expression of VEGF as an upstream regulatory gene [25]. HIF-1*α* and VEGF are positively correlated and play a central role in regulating the RA pathological process of promoting synovial angiogenesis [26] (Figure 5).

**Figure 6:**
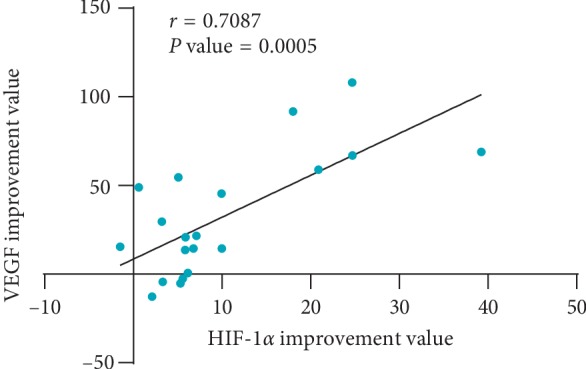
Relationship between the improvement values of HIF-1*α* and VEGF in the treatment group.

We mainly selected HIF-1*α* and VEGF as targets for moxibustion treatment of RA to explore the effect of moxibustion on the HIF-1*α*/VEGF pathway in RA patients. Our study showed that HIF-1*α* and VEGF were significantly reduced after treatment and superior to the control group. Meanwhile, the improvement values of HIF-1*α* and VEGF were positively correlated with each other (Figure 6, performed by GraphPad Prism 8.0.2, GraphPad Prism Software, Inc., San Diego, California, USA). It indicated that moxibustion can reduce the content of VEGF by enhancing the inhibition of HIF-1*α* expression by conventional medicine.

### 4.3. The Moxibustion Strategy

Moxibustion is widely used in clinical treatment of rheumatoid arthritis with obvious analgesic and anti-inflammatory effects [27], but there are fewer clinical studies exploring its mechanisms. In our previous studies, by using high-frequency ultrasound, we observed that moxibustion can enhance the effect of conventional medicine on synovial thickness, blood flow signals, and joint effusion in patients with RA. The improvement scores had a significant correlation with the contents of serum VEGF and IL-1*β* [28]. Combined with our present study, we believe that moxibustion can enhance the effect of conventional medicine, downregulating HIF-1*α*/VEGF contents to inhibit angiogenesis.

ST36 and BL23 are commonly used in acupuncture and moxibustion clinical practice. Animal experiments confirmed that moxibustion of ST36 and BL23 alleviated the cartilage degradation and bone destruction in a rabbit model of RA [15]; electroacupuncture on ST36 and GB39 inhibits synovial angiogenesis in a rat model of adjuvant arthritis [29]. In our study, moxibustion-stimulated ST36, BL23, and Ashi points can improve clinical symptoms, reducing contents of TNF-*α*, IL-1*β* that revealed the analgesic, anti-inflammatory effects in treating RA.

### 4.4. Limitation

Limitations of this study involve the lack of direct observation for the correlation of HIF-1*α*/VEGF and angiogenesis such as the blood flow signals. Although we have determined the relationship between blood flow signals and the contents of serum VEGF and IL-1*β*, as well as the relationship of VEGF improvement value and HIF-1*α* improvement value, we should conduct a further study on the relationship of angiogenesis and HIF-1*α*/VEGF expression.

## 5. Conclusions

The results demonstrated that moxibustion enhanced the anti-inflammatory and analgesic effects of conventional medicine and can enhance the effect of conventional medicine, downregulating HIF-1*α*/VEGF contents to inhibit angiogenesis.

## Figures and Tables

**Table 1:** Baseline characteristics.

	Treatment group (*n* = 20)	Control group (*n* = 17)	*P* value
Characteristic			
Gender, male/female	1/19	2/15	0.452^*∗*^
Age (y), mean (SD)	46.85 (11.30)	49.41 (10.37)	0.48^†^
Disease duration (y), mean (SD)	5.44 (4.05)	6.34 (5.11)	0.55^†^
Clinical symptoms			
VAS score, mean (SD)	6.60 (1.60)	6.71 (1.57)	0.84^†^
Morning stiffness score, mean (SD)	3.60 (1.79)	3.89 (1.11)	0.56^†^
Tenderness index, mean (SD)	2.79 (1.43)	2.77 (1.38)	0.77^†^
Swelling index, mean (SD)	2.67 (1.84)	2.72 (1.64)	0.42^†^
DAS28 score, mean (SD)	6.43 (1.23)	6.87 (0.96)	0.25^†^
RA-associated serum markers			
ESR (mm/60 min), mean (SD)	54.75 (30.94)	57.76 (31.77)	0.87^Δ^
CRP (mg/L), mean (SD)	15.04 (30.18)	22.14 (30.23)	0.13^Δ^
RF (IU/ml), mean (SD)	167.56 (192.89)	185.81 (213.04)	0.58^Δ^

^*∗*^ *P* value by *X*^2^ test. ^†^*P* value by independent samples *t*-test. ^Δ^*P* value by Mann‐Whitney *U* test.

**Table 2:** Clinical Symptoms and RA-associated serum markers.

Outcome measure	Treatment group (*n* = 20)	Control group (*n* = 17)	*P* value
Clinical symptoms			
VAS score, mean (SD)	3.10 (1.52)	5.88 (1.69)	<0.001^†^
Morning stiffness score, mean (SD)	2.00 (1.30)	3.41 (1.37)	0.003^†^
Tenderness index, mean (SD)	1.56 (0.30)	2.62 (0.26)	<0.001^†^
Swelling index, mean (SD)	1.40 (0.38)	2.44 (0.38)	<0.001^†^
DAS28 score, mean (SD)	4.92 (1.18)	6.32 (1.02)	0.001^†^
RA-associated serum markers			
ESR (mm/60 min), mean (SD)	37.20 (28.20)	38.53 (23.17)	0.831^△^
CRP (mg/L), mean (SD)	5.61 (6.90)	6.83 (4.95)	0.070^△^
RF (IU/ml), mean (SD)	120.15 (154.60)	158.38 (189.37)	0.392^△^

^†^ *P* value by independent samples *t*-test. ^△^*P* value by Mann–Whitney *U* test.

**Table 3:** Contents of TNF-*α*, IL-1*β*, VEGF, and HIF-1*α*.

Outcome measures	Treatment group (*n* = 20)	Control group (*n* = 17)	*P* value
TNF-*α* (pg/mL), mean (SD)			
Baseline	30.23 (15.08)	32.27 (16.51)	0.70
Posttherapy	21.53 (13.70)	30.90 (14.14)	0.049
IL-1*β* (pg/mL), mean (SD)			
Baseline	30.16 (15.65)	36.01 (14.87)	0.25
Posttherapy	20.53 (12.88)	30.28 (13.81)	0.033
VEGF (pg/mL), mean (SD)			
Baseline	106.92 (50.69)	126.43 (55.77)	0.273
Posttherapy	65.51 (32.82)	93.36 (45.42)	0.038
HIF-1*α* (pg/mL), mean (SD)			
Baseline	32.92 (18.46)	36.13 (18.60)	0.60
Posttherapy	22.77 (13.10)	33.29 (17.11)	0.048

*P* value by independent samples *t*-test.

## Data Availability

The data used to support the findings of this study are included within the article.
